# Observations on the Effects of Adrenaline

**Published:** 1914-04

**Authors:** 


Observations on the Effects of Adrenaline.—Malcolm Donaldson believes that this drug, while often restoring a pulse previously too feeble to be detected, does so at the expense of injury to the heart. His observations were conducted on healthy normal adults and on several patients apparently needing the drug. Adrenaline was given subcutaneously in doses of three to five minims of the usual one to 1000 solution. The observations were controlled by injections of similar amounts of normal saline solution. In seven normal persons, subjective symptoms noted after the adrenaline were so marked that not one patient was willing to undergo any more observations. Symptoms of which they complained were chiefly breathlessness and a sensation of cold. The former was often extremely distressing; the latter was so intense in one of the patients as to lead to a severe rigor. In normal persons as well as in patients, there was usually blanching of the skin. Blood pressure rose from fifteen to forty-five mm. in normal persons; the pulse rate from fifteen to fifty-two beats a minute. Symptoms usually passed off within a comparatively short time. Donaldson believes that even the subcataneous use of adrenaline is associated with considerable danger, although all of his cases ended favorably.

